# Evaluation of the Quality Changes in Three Commercial Pastourma Samples during Refrigerated Storage Using Physicochemical, Microbiological, and Image Analyses Combined with Chemometrics

**DOI:** 10.3390/foods13071017

**Published:** 2024-03-26

**Authors:** Eftichia Kritsi, Georgia Ladika, Natalia A. Stavropoulou, Marianna Oikonomakou, Alexandros-George Ioannou, Paris Christodoulou, Spyridon J. Konteles, Dionisis Cavouras, Vassilia J. Sinanoglou

**Affiliations:** 1 Laboratory of Chemistry, Analysis & Design of Food Processes, Department of Food Science and Technology, University of West Attica, Agiou Spyridonos, 12243 Egaleo, Greece; ekritsi@uniwa.gr (E.K.); gladika@uniwa.gr (G.L.); nstavropoulou@uniwa.gr (N.A.S.); fst19684070@uniwa.gr (M.O.); alioannou@uniwa.gr (A.-G.I.); pchristodoulou@uniwa.gr (P.C.); skonteles@uniwa.gr (S.J.K.); 2 Department of Biomedical Engineering, University of West Attica, Agiou Spyridonos, 12243 Egaleo, Greece; cavouras@uniwa.gr

**Keywords:** pastourma, image analysis, color, texture, attenuated total reflection–Fourier-transform infrared spectroscopy, microbiological analysis, statistical analysis

## Abstract

Despite the inherent stability of dried and cured products, such as pastourma, appropriate refrigeration remains essential for preserving their optimal characteristics. This study explored quality and safety characteristics in lamb, beef, and buffalo pastourma during 16-day refrigeration storage after package opening. The comprehensive approach employed Attenuated Total Reflection–Fourier-Transform Infrared (ATR-FTIR) spectroscopy, colorimetry, and image analysis, alongside physicochemical and microbiological analyses, to shed light on these alterations. The findings reveal a reduction in textural uniformity and color vibrancy (fading reds and yellows) across all samples during storage, with lamb pastourma exhibiting the most pronounced effects. Notably, image analysis emerged as a powerful tool, enabling the accurate classification of samples based on storage duration. Additionally, significant variations were observed in moisture content, hue angle, firmness, and TBARS levels, highlighting their influence on pastourma quality. The study documented a gradual decrease in lactic acid bacteria and aerobic plate count populations over time. ATR-FTIR spectra’s interpretation revealed the presence of lipids, proteins, carbohydrates, and water. Protein secondary structures, demonstrably influenced by the meat type used, exhibited significant changes during storage, potentially impacting the functional and textural properties of pastourma. Overall, the findings contribute to a deeper understanding of pastourma spoilage during storage, paving the way for the development of improved preservation and storage strategies.

## 1. Introduction

Pastourma, also known as pastirma, a traditional dry-cured meat product, has gained international recognition in the culinary traditions of many regions for its distinct flavor profile and cultural significance, including the Middle East, Central Asia, and the Balkans [1]. The exact origins of pastourma are not definitively documented, but it is thought to have evolved from earlier preservation techniques used in the region. One of the predecessors of pastourma is Apoktin, a Byzantine air-dried salted meat, which could be made from various types of meats, including pig, goat, wild goat, sheep, billy goat, fish, or cuttlefish (historyofgreekfood.eu). Basturma or pastirma is also reported in ancient Armenian and Turkish cuisine. Traditionally, pastourma is prepared in approximately three to four weeks, and its production involves the selection of premium cuts of meat mainly obtained from certain parts of beef and buffalo carcasses [2]. Also, camel, goat, and lamb meats can serve as alternative options in the processing of pastourma [3]. Particularly, in the preparation of pastourma, the entirety of the muscle undergoes trimming to eliminate external fat and connective tissue. Subsequently, the meat is subjected to a dry-curing process employing a curing mixture composed of common salt and sodium nitrite. This procedure is followed by drying, pressing, and coating with an edible paste known as çemen [4].

Çemen paste, a traditional natural coating material, is prepared by mixing fenugreek (*Trigonella foenum graecum* L.) seed flour, freshly crushed garlic, red pepper powder, and an adequate amount of water [5]. The application of çemen paste imparts to pastourma its unique sensory profile, characterized by a distinct appearance, color, texture, taste, and aroma. Also, it serves as a protective barrier against microbial contamination, impedes air exposure, delays spoilage processes, and aids in preventing excessive moisture loss [6,7,8].

To date, a limited number of studies related to the parameters affecting the quality [4,9], composition [10,11], production [12], and shelf-life of [7,13,14] pastourma have been carried out. The surveys mainly focus on determining the effect of different storage temperatures and modified atmosphere packaging (MAP) on the physicochemical and microbiological properties of pastourma samples [7,13,14] and the provision of qualitative pastourma using novel ingredients (such as raspberry-water extract) for the çemen paste preparation [6,15]. Additionally, the identification of the fatty acid composition and a series of physicochemical and microbiological properties of different pastourma types is confined [16].

Currently, image analysis and Attenuated Total Reflectance–Fourier-Transform Infrared (ATR-FTIR) spectroscopy are two emerging technologies that present the potential to provide rapid and objective assessments of food quality and shelf life. In this context, image analysis can be used to evaluate the appearance of food products, such as color, texture, and shape. Changes in these attributes can be indicative of modifications in the quality of the food product [17,18,19]. Similarly, considering the shelf life of a product, ATR-FTIR spectroscopy can be employed to register the absorbance bands of different compounds, giving a quality assessment of food products [17,18,20].

Hence, the goal of the present study was to assess the changes in the quality and safety of pastourma derived from lamb, beef, and buffalo meat during refrigerated storage and in aerobic conditions after opening the packaging by combining physicochemical methods (water activity, moisture content, color parameters, texture attributes, and secondary oxidation products), ATR-FTIR spectroscopy, microbiological analysis, and image analysis. Moreover, discriminant and regression analyses were employed to identify meaningful patterns in the data. Predictive models were then constructed using the image analysis features to forecast the storage days for the pastourma samples. The overall aim was to establish the storage period during which pastourma retains its quality, safety, and suitability for consumption under typical domestic storage conditions.

## 2. Materials and Methods

### 2.1. Sampling

Three types of vacuum-packed sliced (of 2 mm thickness) commercial pastourma, made from lamb, beef, and buffalo, were purchased from a Greek meat products company. For each pastourma type, seven packages of 10 slices per package, with the same expiration date (±3 days) and remaining shelf life before opening (two months), were bought. All pastourma slices were placed into polyethylene containers that were impermeable to moisture, closed, and then stored at 6.0 ± 0.5 °C in a domestic refrigerator (60 ± 2% relative humidity). The temperature was checked using a digital thermometer (measuring range 0–100% RH, −40 to +80 °C, ±0.3 °C accuracy) (mini TH: Humidity and Temperature Data Logger, CAMAR Elettronica, Carpi, Italy). The samples were analyzed for a total of 16 days (days 1, 4, 8, 11, 14, 16). The supply of the samples was made four days before the start of the experimental process, and day 1 refers to the opening of the packages. For each experiment/day, eight (8) slices were utilized per pastourma type.

### 2.2. Image Acquisition

Pastourma samples were photographed digitally with a Sony DSCW800/B (IXUS 100 IS) digital camera (Sony Europe Limited, Edinburgh, UK) positioned at a distance of 15 cm from the surface of the pastourma sample following the procedure outlined by Sinanoglou et al. [18]. For each image, three color features and fifteen textural features were computed from the colored and grayscale versions of the images, respectively.

### 2.3. Discriminant Analysis

**Figure 1:**
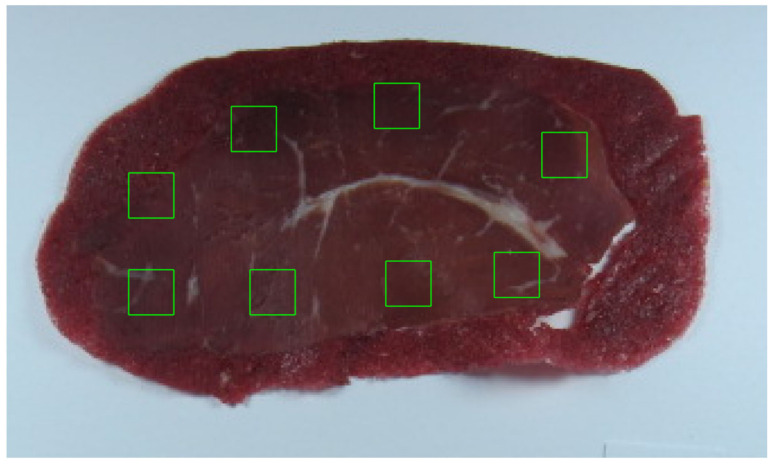
Selection of rectangular regions of interest from the buffalo pastourma image.

Discriminant analysis was utilized to examine alterations in pastourma image texture over a 16-day storage period, aiming to determine the storage days based on pastourma texture features. Initially, pastourma slices were photographed on predetermined measurement days (storage days 1, 4, 8, 11, 14, 16). Employing specialized software, eight color rectangular images (regions of interest or ROIs) (Figure 1) were selected from each pastourma’s photographed slice (Figure 1). Subsequently, three features were calculated from color images, and fifteen textural features (eighteen in total) were generated from the grayscale version of each ROI. The mean feature values were computed from the eight pastourma slice ROIs.

Each pastourma slice was then represented by an 18-feature vector. Six classes, corresponding to the six measurement days, were formed, with each class containing the feature vectors of eight pastourma slices photographed on the respective measurement day. These classes were employed in discriminant and statistical analyses.

For discriminant analysis, machine learning (ML) classifiers were used. These classifiers, available in the Python programming language library sklearn, included Classification and Regression Decision Tree, K-Nearest Neighbor, Linear Discriminant Analysis, Logistic Regression, Multi-Layer Perceptron, Naïve Bayesian, Nearest Centroid, Perceptron, Random Forest, and Support Vector Machines. The accuracy of a classifier in assigning pastourma image ROIs to the correct storage day was evaluated by assessing labeled pastourma slice image ROIs.

To construct the ML system, one classifier was chosen, and a precision evaluation method (leave one out or LOO) was utilized. The ML system’s design precision was tested using different classifiers, normalizing features to zero mean and unit standard deviation and exploring all possible feature combinations. Dimensionality reduction involved compacting each feature combination into two Principal Component Analysis (PCA) components, PCA1 and PCA2, which were then input into the ML system. Two-dimensional scatter plots based on PCA were generated to display the results of the discriminant analysis, showcasing class-separating surfaces.

### 2.4. Determination of Physicochemical Parameters during Pastourma Storage

Pastourma water activity (a_w_) was measured using an a_w_-meter (AquaLab Dew Point Water Activity Meter 4TE, METERGroup, Inc., Pullman, WA, USA).

Moisture content was measured by placing 0.2–0.4 g of pastourma slice on the sample pan of an Electronic Moisture Analyzer (Kern MLS 50-3, KERN & SOHN GmbH, Balingen, Germany).

For the hue (h) angle (in degrees) determination, a tristimulus chromatometer (model CR-400, Minolta, Tokyo, Japan) was used, calibrated with a white standard plate (L*: 97.83, a*: −0.45, b*: +1.88). The measurements were taken at the surfaces of pastourma slices.

Pastourma texture characteristics were measured using a texture analyzer (TA-XTplusC, Stable Micro Systems, Godalming, UK), according to Sinanoglou et al. [18].

### 2.5. Thiobarbituric Acid Reactive Substance (TBARS) Assay

The determination of secondary oxidation products was performed by measuring thiobarbituric acid reactive substances (TBARSs), according to the method described by Papastergiadis et al. [21]. The absorbance was measured at 532 nm using a Spectro 23 Digital Spectrophotometer (Labomed, Inc., Culver City, CA, USA). The TBARS results were expressed as mg of malondialdehyde per 1 kg of pastourma, using the standard curve obtained from standard solutions prepared from 1,1,3,3-tetraethoxypropane with a concentration range from 0.6 to 10 μM.

### 2.6. Fourier-Transform Infrared Spectroscopy with Attenuated Total Reflectance (ATR-FTIR)

The Fourier-Transform Infrared (FTIR) spectra of pastourma samples were captured at room temperature through Attenuated Total Reflectance (ATR) using an IRAffinity-1S FTIR Spectrometer from Shimadzu, Kyoto, Japan. The ATR reference was set at 3284.77 cm^−1^. Spectra for both background and samples were obtained in the range of 4000 to 499 cm^−1^, averaging 20 scans with a resolution of 4 cm^−1^. Subsequently, the FTIR spectra underwent ATR correction, smoothing, normalization, and peak-picking processes. The data processing steps were carried out using LabSolutions IR software (version 2.21) [22].

The amide I region (1600–1700 cm^−1^), as it contains information about the protein’s secondary structure, was smoothed using a nine-point Savizky–Golay function to reduce noise. A baseline correction was then performed to enhance the accuracy of subsequent analysis. The second derivative analysis was used via Origin 8.5 software (OriginLab Corporation, Northampton, MA, USA) to pinpoint peak positions indicating different proteins’ secondary structures in the amide I region. Gaussian functions were used for the curve-fitting analysis of the amide I band. This involved fitting Gaussian curves to the peaks identified in the spectrum. The relative abundance of different secondary structures was determined by analyzing the areas under the deconvoluted peaks. The proportion of proteins’ secondary structures was calculated by dividing the individual deconvoluted peak area by the total amide I peak area and then multiplying by 100 based on the equation below:

(1) $$\frac{Individual\;deconvoluted\;peak\;area}{Total\;amide\;I\;peak\;area}\times 100=Protein\;secondary\;structure\;proportion$$

### 2.7. Microbiological Analyses

Ten (10) g of each sample was placed in a Stomacher filter bag, and 90 mL of sterile buffered peptone water (BPW, Oxoid CM1049, Thermo Fisher Scientific, Waltham, MA, USA) was added. The content of each bag was homogenized with a laboratory blender (Stomacher 400, Seward Ltd., Company, West Sussex, UK) for 2 min. Serial decimal dilutions were made with buffered peptone water, and then, the proper quantity of each dilution was plated. The aerobic plate counts (APCs) were enumerated on the Plate Count Agar (PCA, HiMedia, Mumbai, India) plates incubated at 30 °C for 48 h, and the lactic acid bacteria on MRS plates (De Man, Rogosa Sharpe, Merck, Darmstadt, Germany) were incubated at 37 °C for 48 h anaerobically (BBL GasPak jar anaerobiosis system). For yeast counting, 0.1 mL of the serial dilutions was spread plated on Potato Dextrose Agar (PDA, Merck, Germany) and incubated at 20 °C for 5 days. Counts of microorganisms were determined as log cfu/g.

### 2.8. Statistical Analysis

The pastourma features derived from image analysis across the storage days underwent statistical analysis using the non-parametric Mann–Whitney–Wilcoxon test for two classes. The analysis was performed using the Python scipy.stats library (https://docs.scipy.org/doc/scipy/tutorial/stats.html, accessed on 10 January 2024).

The prediction of the actual storage days for pastourma samples involved the application of multiple regression. This was achieved through the utilization of the Gradient Boosting Regressor model and the K-fold evaluation method. The objective was to forecast the duration of storage by analyzing both the images extracted and the physicochemical features of the samples.

The ATR-FTIR, as well as the physicochemical and microbiological results, underwent analysis using a one-way ANOVA and subsequent post hoc analysis (Tukey Honest Significant Difference (HSD) post hoc test) in SPSS (IBM SPSS Statistics, version 29.0, Chicago, IL, USA) at a significance level of 95% (*p* < 0.05).

## 3. Results and Discussion

### 3.1. Image Texture Evaluation of Pastourma Samples during Storage

**Figure 2:**
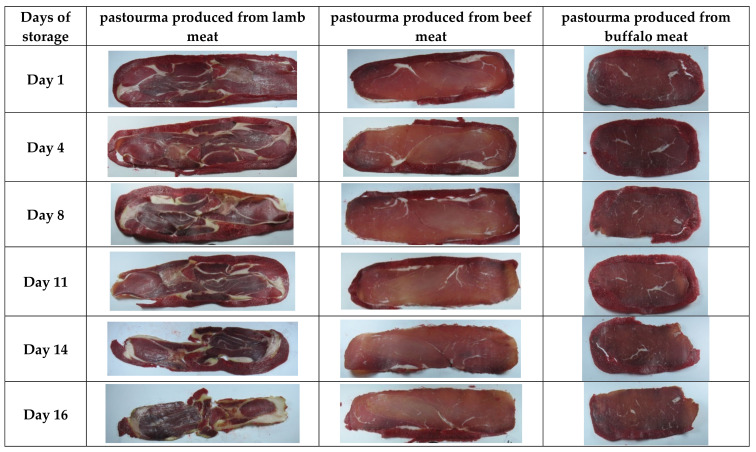
Illustrative images of pastourma slices during a 16-day refrigerator storage (days 1, 4, 8, 11, 14, and 16).

Image analysis offered a rapid and non-destructive method of assessing the quality and stability of pastourma slices produced from lamb, beef, and buffalo meat during refrigerator storage. The progressive changes in the pastourma slices’ appearance and color over a 16-day storage period, as depicted through image analysis, are illustrated in Figure 2.

To evaluate the pastourma samples’ quality and stability through the variations in their images and textures during refrigerator storage, machine learning techniques were utilized to analyze the computed features derived from both colored and grayscale images of the pastourma slices.

**Figure 3:**
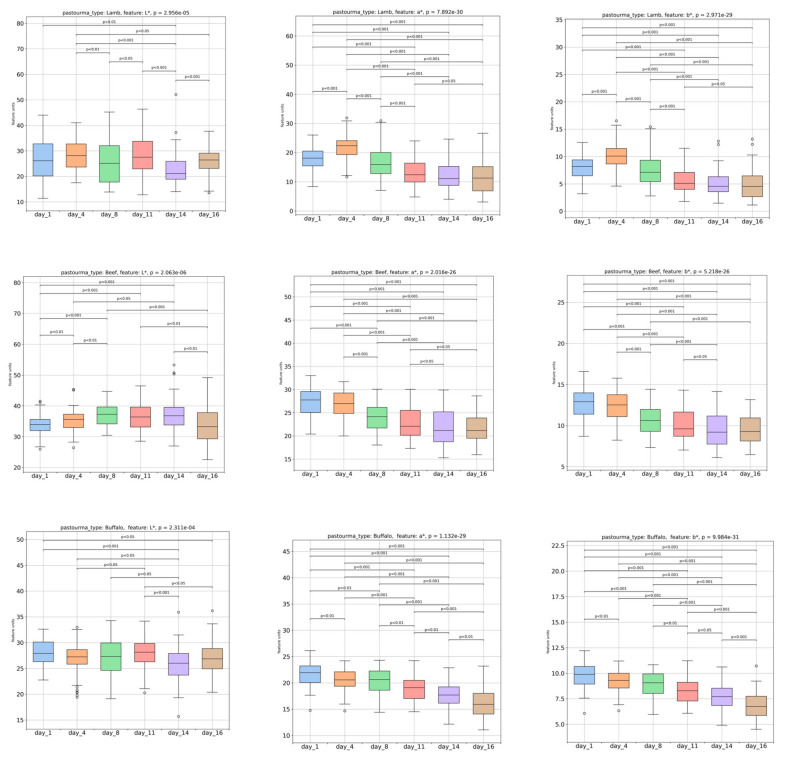
Color parameters (L*, a*, and b*) resulting from image analysis of lamb, beef, and buffalo pastourma over a storage period of 1, 4, 8, 11, 14, and 16 days.

Figure 3 illustrates the variations in the color parameters L* (lightness), a* (redness–greenness), and b* (yellowness–blueness) extracted from the colored images of the pastourma slices. The L*, a*, and b* color parameters were significantly (*p* < 0.05) affected by the pastourma type and presented their higher values in beef pastourma and lower values in lamb pastourma. The redness of pastourma slices can be influenced by various factors, including production and curing conditions and duration, curing agents (colorants, nitrate, and salt), storage conditions, çemen composition, and microbiological stability, as indicated by Aksu et al. [23]. In all pastourma samples, the L* values showed significant fluctuations during refrigerator storage. Specifically, in buffalo pastourma, there was a significant decrease in L* values during storage, which suggests that the buffalo pastourma became darker over time compared to the other types of pastourma. During storage, the color parameters a* and b* of all pastourma types showed a gradual and significant decrease, displaying a linear relationship between them, consistent with the findings of other studies [16,23]. The decrease in a* and b* values was more distinct on the 8th day for pastourma produced from lamb and beef and on the 11th day for the buffalo one. This indicates that all pastourma samples became less red and less yellow over time. The decrease in a* and b* values could be the result of the conversion of myoglobin (red color) to metmyoglobin (brown color) [4]. Moreover, according to a previous study by our research team [17], the changes in color parameters of pork and turkey hams during refrigerator storage were attributed to the dehydration of the ham slices’ surface and the oxidative degradation of lipids.

**Figure 4:**
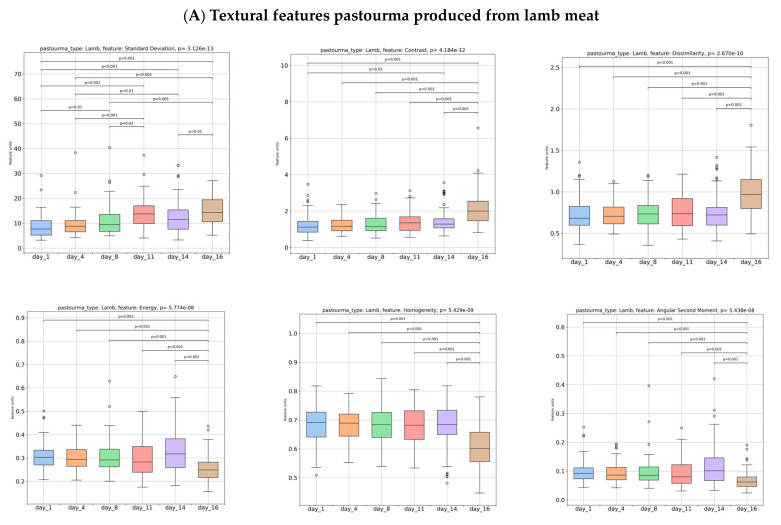

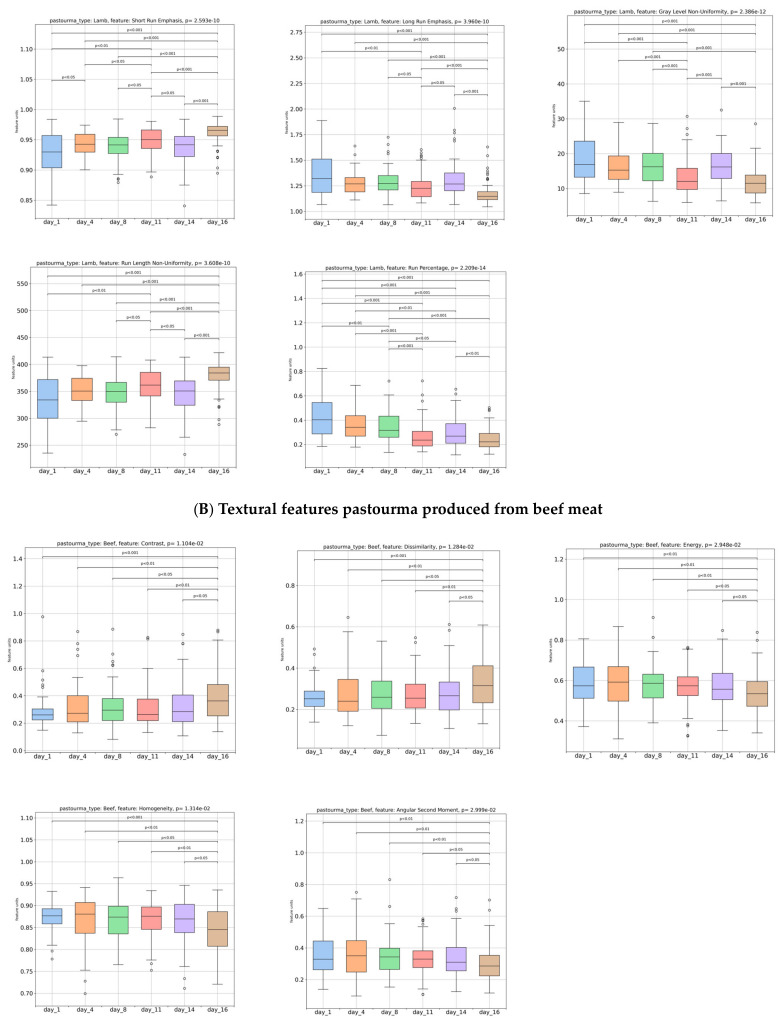

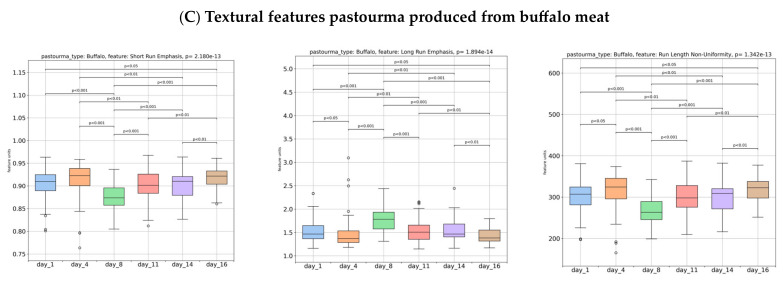
Computed features [standard deviation, contrast, dissimilarity, short-run emphasis (SRE), run length non-uniformity (RLN), energy, homogeneity, angular second moment (ASM), long-run emphasis (LRE), gray level non-uniformity (GLN), and run percentage (RP)], resulting from grayscale images of lamb, beef, and buffalo pastourma over a storage period of 1, 4, 8, 11, 14, and 16 days.

Figure 4 illustrates the variations in the textural features extracted from the grayscale images of pastourma slices. In pastourma produced from lamb meat (Figure 4A), with increasing storage time, the textural features standard deviation (variation from the average value), contrast (degree of changes in pixel intensity values within a picture), dissimilarity (textural image variation), short-run emphasis (occurrence of short, consecutive runs of similar pixel intensity values within an image), and run length non-uniformity (variations in the lengths of runs of pixels with similar intensity values within an image) significantly (*p* < 0.05) increased, while energy (the uniformity or homogeneity of pixel intensities in an image), homogeneity (the similarity of neighboring pixel intensities in an image), angular second moment (the overall homogeneity of an image), long-run emphasis (longer consecutive runs of pixels with similar intensity values in an image), gray level non-uniformity (the variability in the distribution of gray levels or pixel intensities in an image), and run percentage (spatial distribution of texture features across the image and overall texture complexity) decreased (*p* < 0.05). Therefore, as the storage time progressed, lamb pastourma images became more distinct in terms of the distribution among the gray levels of the image structures, and the internal structure of pastourma lost its uniformity and orderliness and became less consistent or more fragmented, especially on day 16.

In pastourma produced from beef meat (Figure 4B), with increasing storage time, the textural feature contrast and dissimilarity significantly (*p* < 0.05) increased, while energy, homogeneity, and angular second moment decreased (*p* < 0.05). Regarding the pastourma produced from buffalo meat (Figure 4C), the textural feature short-run emphasis and run length non-uniformity significantly (*p* < 0.05) increased, while long-run emphasis decreased (*p* < 0.05) during storage. The results in beef and buffalo pastourma were less pronounced than those in lamb pastourma, indicating that the increase in textural structure dissimilarity during refrigerator storage affected fewer textural features. Generally, textural feature alterations resulting from image changes on the surface of meat products could be used as a tool to assess their quality during storage [17].

Monitoring these textural features through imaging techniques can provide valuable insights into the quality changes that occur in meat products over time. For instance, alterations in texture may indicate changes in moisture content, fat distribution, protein denaturation, or the development of undesirable characteristics like spoilage or microbial growth. By assessing these textural changes, food scientists and producers can better understand how a meat product is evolving during storage and make informed decisions about its quality, shelf life, and potential consumer acceptability. This information can also be used to optimize storage conditions or develop preservation strategies to maintain the desired quality of meat products for longer periods of time.

As a further step, discriminant analysis was employed, using the Classification and Regression Decision Tree classifier, the PCA feature reduction method, and the textural features extracted from the colored and grayscale images of the pastourma samples to group the pastourma samples based on two factors—meat type (lamb, beef, and buffalo) and storage days (day 1 and day 16)—and to identify the most effective combinations of textural features that can discriminate, employing the pastourma types and different storage days. This approach could help in quality assessment and classification and potentially in developing predictive models for the behavior of pastourma during storage.

**Figure 5:**
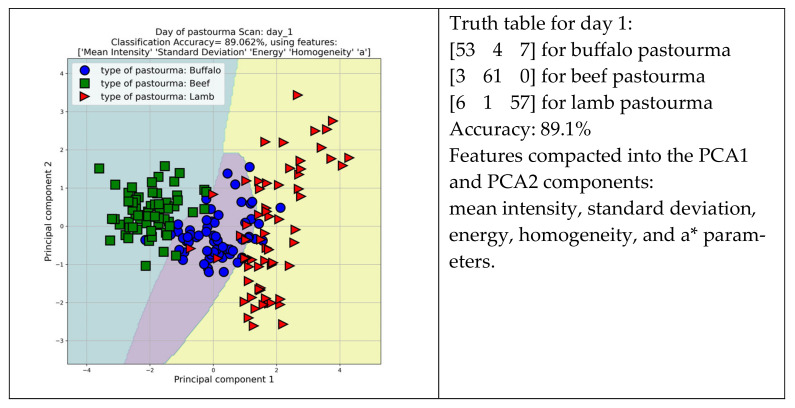

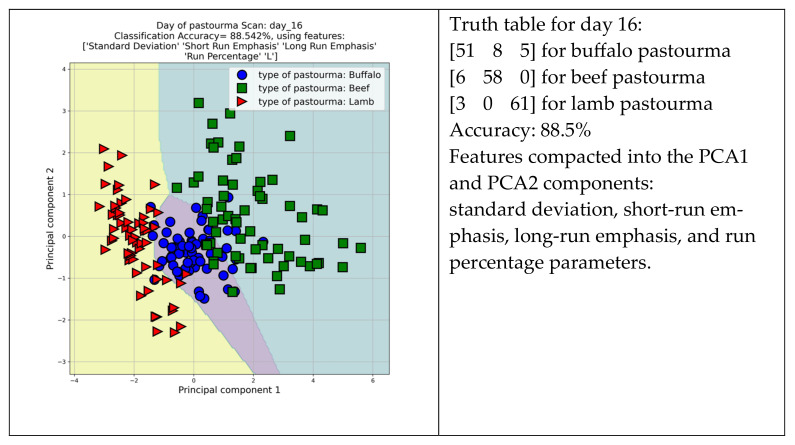
Scatter diagrams presenting the discrimination among lamb, beef, and buffalo pastourma samples on day 1 and day 16.

Satisfying discrimination among lamb (red triangles), beef (green squares), and buffalo (blue circles) pastourma samples on day 1 and day 16 was achieved with 89.1% and 88.5% accuracy, respectively (Figure 5). The features combination employed for discrimination on day 1 were mean intensity, standard deviation, energy, homogeneity, and a* parameters. This five-feature combination designed a high-performance pattern recognition system that correctly classified 53, 61, and 57 buffalo, beef, and lamb pastourma slices, respectively, out of 64 slices. Moreover, the discrimination on day 16 was achieved according to the combination of features: standard deviation, short-run emphasis, long-run emphasis, and run percentage. This four-feature combination correctly classified 51, 58, and 61 buffalo, beef, and lamb pastourma slices, respectively, out of 64 slices.

**Figure 6:**
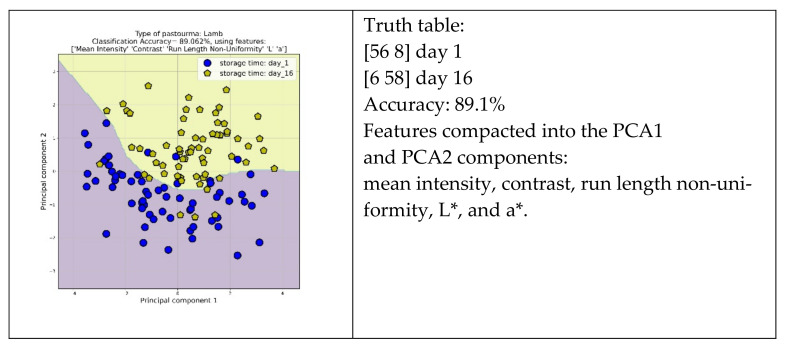

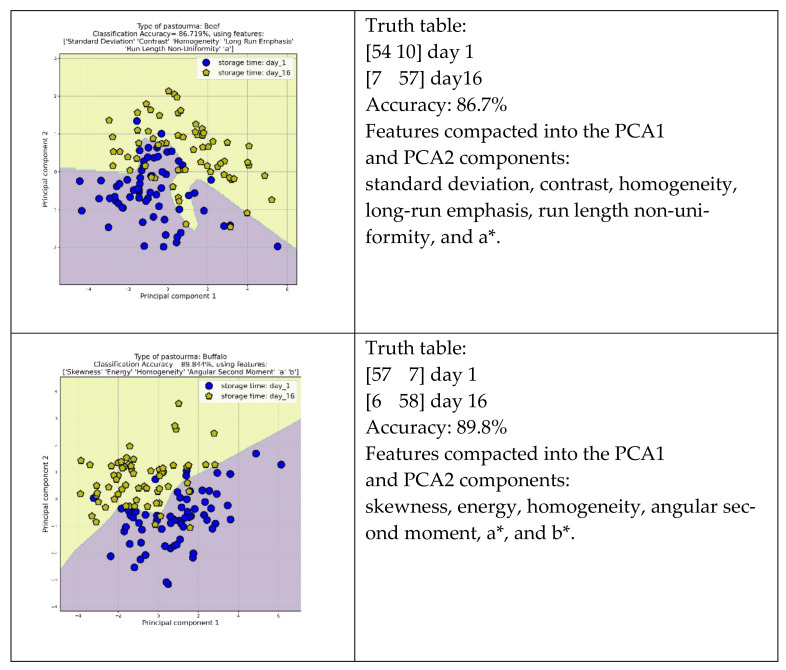
Scatter diagram presenting the discrimination between day 1 and day 16 for lamb, beef, and buffalo pastourma samples.

The scatter diagrams in Figure 6 illustrate the best discrimination between day 1 and day 16 samples of lamb, beef, and buffalo pastourma slices based on certain textural features. These features include mean intensity, standard deviation, contrast, homogeneity, long-run emphasis, run length non-uniformity, skewness, energy, angular second moment, and color-related features, such as L*, a*, and b*. The overall discrimination accuracy ranged at high levels, ranging from 86.7% to 89.8%. This suggests that the selected combination of features provided valuable information for distinguishing between day 1 (blue circles) and day 16 (yellow pentagon) samples for all types of pastourma (lamb, beef, and buffalo). Moreover, the pattern recognition system correctly classified a significant portion of the samples. Specifically, for lamb pastourma, it correctly classified 56 and 58 slices on days 1 and 16, respectively, out of 64 samples. Similarly, for beef pastourma, it correctly classified 54 and 57 slices out of 64 samples for days 1 and 16, correspondingly. For buffalo pastourma, it correctly classified 57 and 58 slices out of 64 samples for days 1 and 16, correspondingly.

**Figure 7:**
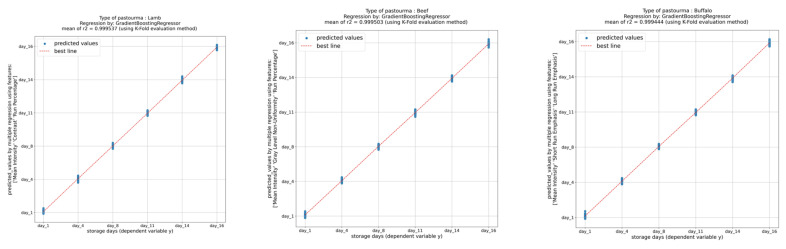
Prediction of storage days from textural features.

Next, a regression analysis was conducted to develop models for predicting the storage days of pastourma samples based on the image-extracted features. The gradient boosting regressor model was chosen for this task. Gradient boosting is a machine learning technique used for regression and classification tasks. It builds multiple decision trees sequentially, where each tree corrects the errors of its predecessor, leading to improved accuracy. The K-fold evaluation method was also employed. In K-fold cross-validation, the dataset is divided into K subsets, and the model is trained and evaluated K times, each time using a different subset as the test set and the remaining subsets as the training set. This method helps in obtaining a more reliable estimate of a model’s performance. The coefficient of determination (R-squared, R^2^) is a measure of how well the regression model fits the observed data. The textural features with the highest coefficient of determination (R^2^ > 0.999) were mean, run percentage (RP), and contrast for lamb pastourma; mean, run percentage (RP), and gray level non-uniformity (GLN) for beef pastourma; and mean, short-run emphasis (SRE), and long-run emphasis (LRE) for buffalo pastourma (Figure 7). Overall, the findings suggest that the gradient boosting regressor model trained on image-extracted features can effectively predict the storage day of pastourma samples with high accuracy.

### 3.2. Storage Effect on the Physicochemical Features of Pastourma Samples

Pastourma samples were evaluated over a 16-day storage period at a controlled temperature of 6.0 ± 0.5 °C by measuring the water activity (a_w_), moisture content (% w/w), color parameter hue, firmness (N), and thiobarbituric acid reactive substances (TBARSs) (Table 1). Monitoring these physicochemical parameters allows for the understanding of how pastourma samples change over time, determining their quality and stability under aerobic refrigerated storage conditions.

The water activity measurements did not show any significant changes for all pastourma samples during the storage period. Specifically, a_w_ values were calculated at 0.90 for lamb pastourma samples, 0.87 for beef samples, and 0.84 for buffalo samples.

The moisture content of pastourma produced from beef meat exhibited the highest (*p* < 0.05) value compared to pastourma produced from lamb and buffalo meat, which showed similar values. This alteration could be attributed to the different animal species and muscle cuts used and variations in the processing methods and time in pastourma production [16]. Moreover, the results of the moisture content of the studied pastourma species are similar to those reported in the literature [16,24,25]. Furthermore, the results indicate that the moisture content (%) of all pastourma samples decreased significantly and progressively over the 16-day storage period. This decrease was observed consistently across all samples. Interestingly, the lowest moisture content values were reached between the 8th and 11th days of the experiment. This finding suggests that moisture loss that occurred in the pastourma samples was possibly due to the refrigerator storage.

The hue angle (h) color parameter was significantly (*p* < 0.05) affected by the pastourma type, presenting its higher value in beef pastourma and lower value in lamb pastourma. Refrigerator storage caused different changes in hue values depending on the pastourma type. The hue values of lamb pastourma significantly (*p* < 0.05) decreased on the 11th day of storage, followed by an insignificant decrease until day 16. The hue values of beef pastourma decreased significantly (*p* < 0.05) at the end of the storage period, whereas the values of buffalo pastourma remained statistically unchanged throughout the storage period. According to Table 1, beef and buffalo pastourma samples maintained their red/orange color, whereas lamb pastourma developed a red/brown color. The decrease in the h value observed during the refrigerator storage of pastourma can be attributed to the conversion of oxymyoglobin to metmyoglobin, which has a brownish color as a result of oxidation processes, a finding that has also been found by other studies [26].

Lamb and buffalo pastourma slices were characterized by significantly higher (*p* < 0.05) firmness (N) values compared to beef slices. The textural properties of pastourma can be greatly influenced by the type and composition of meat used and the methods applied in its production, including curing, pressing, and drying [2]. The firmness of lamb, beef, and buffalo pastourma slices increased significantly and gradually between the 8th and 11th day, on the 8th day, and between the 4th and 11th day, respectively. Textural changes in meat products during cold storage are attributed to various factors, including enzymatic activity, changes in moisture content, and reactions within food polymers that result in hardening. Specifically, changes in moisture content can alter the structural integrity of food components and affect their texture. Moreover, an increase in firmness can be attributed to changes in protein conformation and unfolding, which affect the formation of the product’s three-dimensional network. Additionally, alterations in the size of fat particles and water content can also contribute to this phenomenon [27]. Furthermore, as the meat product undergoes storage, there may be a reduction in the amount of water that is tightly bound or immobilized within the food matrix, which can lead to changes in the overall texture and hardness of the product [28].

According to the results presented in Table 1, significant variations in thiobarbituric acid reactive substance (TBARS) values were observed among different types of pastourma, with lamb pastourma exhibiting the highest values (*p* < 0.05). The multiple TBARS values observed in lamb pastourma may be attributed to lipid oxidation processes occurring during its production. Similar TBARS values to lamb, beef, and buffalo pastourma have been reported by Gençcelep et al. and Gök et al. [7,25]. Moreover, the TBARS values of lamb pastourma slices showed a significant increase (*p* < 0.05) at the end of the storage period. In contrast, the TBARS values of beef and buffalo pastourma demonstrated a progressive and significant increase from the 4th day of storage until the end of the storage period.

Conclusively, the results of the a* parameter, hue angle, and TBARS values, especially in lamb pastourma, confirm that the combined effects of oxymyoglobin oxidation and lipid oxidation can lead to discoloration of meat, resulting in a decrease in redness and hue value, as reported by Chaijan [29].

### 3.3. Attenuated Total Reflection–Fourier-Transform Infrared (ATR-FTIR) Spectra of Pastourma Samples during Storage

The ATR-FTIR spectra bands for all pastourma samples range from 3300 to 500 cm^−1^. Analyzing the absorption bands provides information regarding the presence of lipids, proteins, peptides, carbohydrates, and water. Table 2 displays the spectral absorbance bands (intensities) observed in pastourma derived from lamb, beef, and buffalo meat throughout the storage period. The primary absorptions identified, along with their corresponding vibrations, are detailed below. Additionally, an assessment of the key findings pertaining to the pastourma samples is presented.

The intensity of the band ranging from 3500 to 3300 cm^−1^, attributed to O-H stretching vibrations in polysaccharides, particularly glycogen, remained statistically unchanged. Meanwhile, the intensity in the region of 3290–3287 cm^−1^, primarily associated with O-H stretching vibrations of water [30], exhibited a notable (*p* < 0.05) decrease during the storage period for pastourma derived from lamb, beef, and buffalo. This observation aligns with the results of the moisture content analysis. The intensities of the asymmetric stretching vibrations of the C-H in the methyl group of lipids at 2960–2950 cm^−1^ [31] exhibited negligible variations. In contrast, the intensities in the methylene group of lipids at 2922 cm^−1^ showed a significant (*p* < 0.05) increase, particularly after the fourth day of storage. The intensities of the symmetric stretching vibrations of C-H in both the methyl and methylene groups of lipids, observed at 2877–2870 and 2854 cm^−1^, respectively, displayed significant (*p* < 0.05) fluctuations in almost all pastourma samples throughout the storage period. It is crucial to note that lamb pastourma exhibited markedly higher absorptions at 2922 and 2854 cm^−1^ compared to the other pastourma samples, confirming its elevated fat content.

The intensities of the carbonyl (C=O) stretching vibrations associated with cholesterol esters and triglycerides, as well as aromatic esters at 1743 and 1728 cm^−1^, respectively [31,32,33], exhibited opposing changes in different types of pastourma, as shown in Table 3. The occurrence of aromatic esters on the 14th day of storage in lamb pastourma could possibly be attributed to the potential degradation of its lipids [34]. The intensities observed at 1627, 1541, and 1314 cm^−1^, representing the robust absorption band of C=O stretching vibration (amide I band), the combination of C-N stretching and N-H bending vibrations (amide II band), and the combination of N-H bending, C-N stretching, and O=C–N bending vibrations (amide III band) in peptides and proteins [17,31], displayed minimal significant or non-significant alterations. The absorbances observed at 1450–1452 and 1392–1400 cm^−1^ are linked to the methyl and methylene bending vibrations (wagging, twisting, and scissoring) of proteins [17,31]. Notably, the intensity at 1450–1452 cm^−1^ demonstrated noteworthy fluctuations, indicating a significant decrease in lamb and buffalo pastourma while exhibiting an increase in beef pastourma.

The absorption bands at 1238–1242 and 1078–1083 cm^−1^ signify the asymmetric and symmetric stretching of PO_2_ found in nucleic acids, phospholipids, or phosphorylated proteins in meat products [17,31,35]. Furthermore, in the region 1172–1157 cm^−1^, the stretching vibrations of the CO and C–OH groups associated with amino acids, proteins, and carbohydrates are observed [31,35]. Lastly, at 1060 cm^−1^, C–O stretching vibrations in nucleic acids and polysaccharides, predominantly glycogen, are evident [31].

ATR-FTIR has been widely recognized as a robust analytical method for examining protein secondary structures and local conformational alterations. As per the existing literature, the distribution of protein secondary structures in foods can undergo changes due to a variety of processes, including radiation, hot air roasting, microwave processing, freeze-drying, brine salting, cooking, food production, ripening, or/and during food transport and storage [36,37,38].

**Figure 8:**
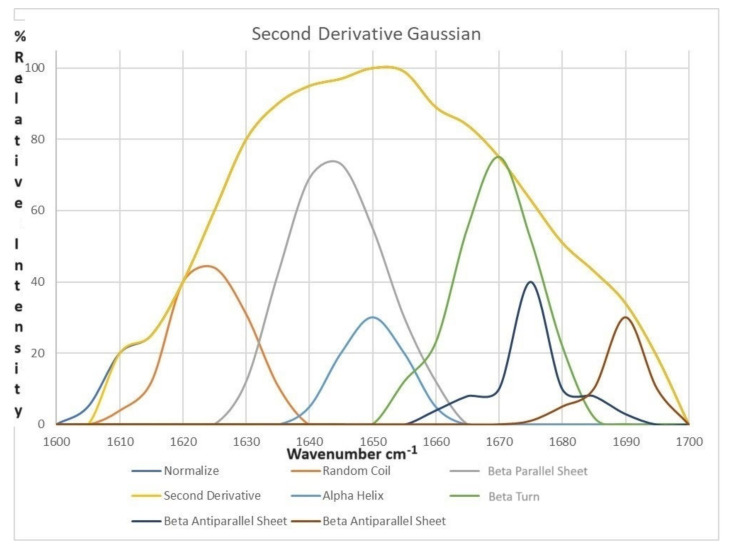
Second derivatives of the ATR-FTIR spectrum of amide I region and its Gaussian components for the beef pastourma at day 16.

Therefore, the amide I region of the pastourma samples’ spectra was examined to interpret the protein secondary structures, aiming to assess the impact of storage on the conformation of pastourma protein secondary structures. More specifically, the amide I region, from 1600 to 1700 cm^−1^, was isolated to calculate the second derivative, as presented in Figure 8. The quantified percentages for the *β*-parallel sheet, random coil, *α*-helix, *β*-turn, and *β*-antiparallel sheet secondary structures of proteins observed in all pastourma samples on both day 1 and day 16 of the storage period are presented in Table 3.

According to the findings in Table 3, it appears that initially, in all pastourma samples, the major secondary structures were *β*-turn and *β*-antiparallel sheets, collectively constituting approximately 61–71% of the total secondary structure. Notably, the *β*-turn structure predominated in pastourma derived from lamb meat, while the *β*-antiparallel sheet was more prominent in pastourma produced from beef and buffalo meat. At the end of the storage period, significant differences were observed in the distribution of protein conformations, which exhibited alterations across different types of pastourma.

More specifically, a significant (*p* < 0.05) increase in the *β*-antiparallel sheet structure, a significant (*p* < 0.05) decrease in the structures *β*-parallel sheet, *α*-helix, and random coil, and a non-statistical (*p* > 0.05) change in the *β*-turn structure were found in the pastourma samples derived from lamb meat at the end of the storage period. The results demonstrate that the *β*-parallel sheet, *α*-helix, and random coil were converted into a *β*-antiparallel sheet during refrigerator storage. This conversion may be related to the fact that the *β*-antiparallel sheet exhibited greater stability than the *β*-parallel sheet because of differences related to chain packing and peptide dipole alignment [39]. Moreover, the distribution of *α*-helix and *β*-sheet structures in proteins is influenced by hydrogen bond stability within and between peptide chains, respectively [40].

Regarding the pastourma samples produced from beef and buffalo meat, the storage process resulted in a significant (*p* < 0.05) increase in *α*-helix and *β*-turn conformations, a significant (*p* < 0.05) decrease in the *β*-antiparallel sheet structure, and a non-statistical change in the *β*-parallel sheet and random coil conformations. In accordance with the above finding, Qian et al. [41] reported that lower temperatures can promote the formation or stabilization of *α*-helix structures in beef myofibrillar protein. Moreover, both *α*-helix and *β*-turn conformations contribute to the overall stability and integrity of protein structures [42]. Furthermore, an increase in their proportions suggests a more ordered and compact arrangement of the protein, which can enhance its stability against denaturation and degradation processes. This increased stability is often desirable in various applications, including food processing, where maintaining protein structure is crucial for product quality and shelf life.

It is interesting to note that the variations in protein secondary structures are dependent on the type of meat used in pastourma production. These alterations ultimately impact the overall quality of the pastourma samples. Indeed, various studies have documented that the refrigerated storage of meat can lead to significant changes in the protein secondary structure, which, in turn, affects, the functional and texture properties of the meat products [43].

### 3.4. Microbiological Analyses

**Figure 9:**
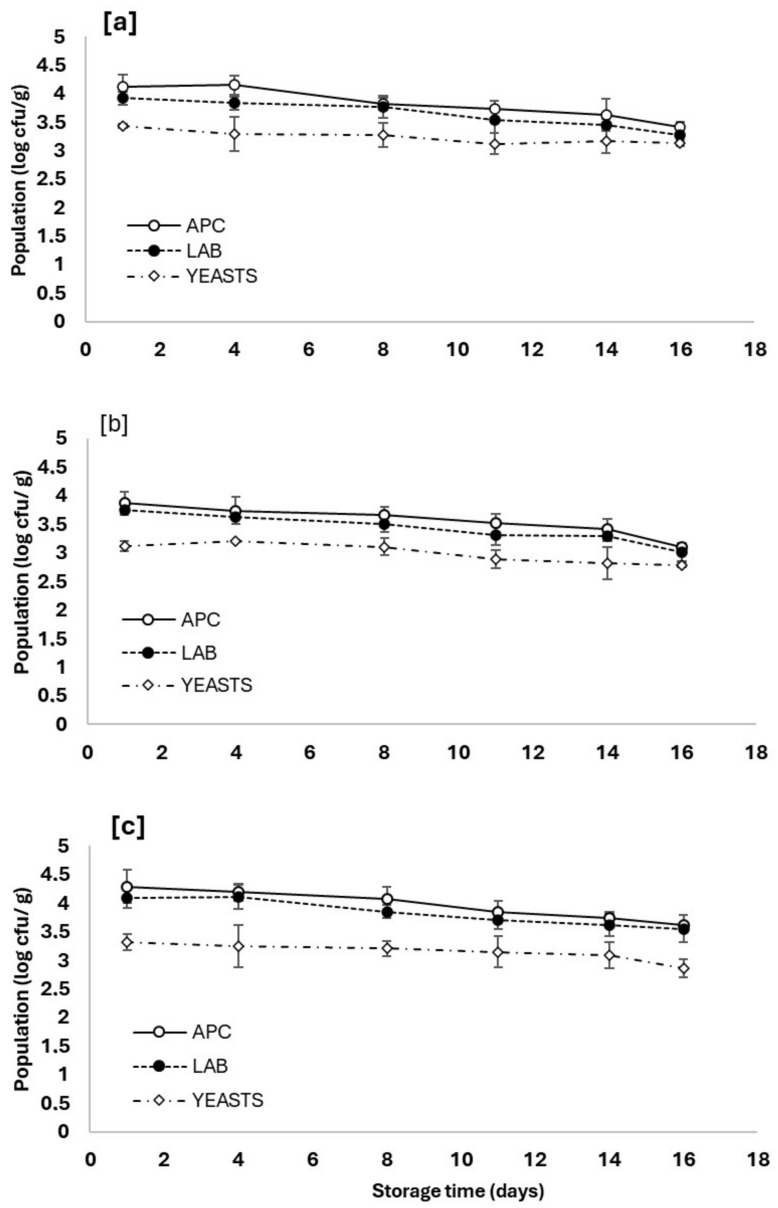
Changes in microbial populations in pastourma samples made of (**a**) lamb, (**b**) beef, and (**c**) buffalo pastourma aerobically stored at 6 °C for 16 days. The populations counted were aerobic plate count (APC), lactic acid bacteria (LAB), and yeasts.

Often, the microbiological characteristics of perishable foods are one of the major indexes of their quality. In Figure 9, the changes in aerobic plate count (APC), lactic acid bacteria (LAB), and yeasts in the three types of open pastοurma samples stored at 6 °C are shown. In all tested samples, the trend of the microorganism counts was identical. The initial bacterial load was approximately 10^4^ cfu/g, while the initial yeast count was about 1 log cycle lower. The initial counts of the LAB in the samples are consistent with previous studies that have reported significant fluctuations in LAB amongst pastourma samples. Aksu and Kaya [44] analyzed 48 market available pastourma samples, and the LAB population was 3.75–7.89 log cfu/g, while more recently, Öz et al. [45] analyzed 14 different samples from the market, and the LAB counts were from 3.30 to 7.90 log cfu/g. There were no statistically significant differences between the APC and LAB across all the samples examined, indicating that the two populations were virtually indistinguishable. This is almost certainly attributable to vacuum packaging, which promotes the growth of facultative anaerobic lactic acid bacteria.

Interestingly, during the domestic storage of the open samples, the population of APC and LAB was slightly decreasing, and on the last day of the storage, both bacterial groups were statistically lower than the initial population. This trend should probably be attributed to the gradual dehydration of the samples, which hinders LAB growth. A similar tendency to decrease the LAB population during dehydration phenomena has been reported by Lorenzo et al. [46] during the production of lacón, a Spanish traditional dry-cured meat product. In contrast, as regards the group of yeasts, no statistically significant change was observed between the 1st and 16th days of preservation. Hence, it can be concluded that during refrigerator storage, the growth rates of the aerobic plate count (APC) and lactic acid bacteria (LAB) in pastourma samples were notably diminished primarily due to the decrease in moisture content.

## 4. Conclusions

The latest study utilized a cohesive analytical strategy, merging non-destructive image analysis with infrared spectroscopy, physicochemical, and microbiological analysis, to evaluate maintaining the quality and safety of pastourma slices during refrigerator storage in aerobic conditions. The most significant findings are outlined as follows:

In terms of the computed features obtained from the colored images of pastourma slices, it was observed that all pastourma samples exhibited a decrease in redness and yellowness over the storage period, with the effect becoming more pronounced between the 8th and 11th days of storage. Furthermore, the changes in textural features derived from the grayscale images of pastourma slices indicated an increase in textural dissimilarity during refrigerator storage across all types of pastourma, with lamb pastourma showing more prominent results.

A consistent decrease in moisture content and a notable increase in firmness (N) values were observed across all pastourma samples, with the moisture content reaching its lowest and firmness its highest values between the 8th and 11th days of refrigerator storage. Additionally, beef and buffalo pastourma samples retained their red/orange hue, while lamb pastourma acquired a red/brown color after the 11th day, attributed to a higher decrease in hue angle. In order to assess the shelf life of refrigerated pastourma samples, thiobarbituric acid reactive substance (TBARS) measurement and microbiological analysis were applied. The results reveal an increase in TBARS values and a decrease in aerobic plate count (APC) and lactic acid bacteria (LAB) counts during storage, which is likely linked to the decrease in moisture content over time.

The interpretation of ATR-FTIR spectra revealed distinctions in lipids, proteins, peptides, carbohydrates, and water content among pastourma samples, both in terms of meat type and storage process. It also confirmed the reduction in moisture content and alterations in the protein and lipid content during refrigerator storage. Interestingly, the distribution of protein secondary structures was found to be dependent on the type of meat used in pastourma production and refrigerator storage.

A high-accuracy classification of pastourma samples based on meat type and days of refrigerator storage was successfully attained through the PCA analysis of image-extracted features. Additionally, a gradient boosting regressor model was utilized to accurately predict the quality of pastourma concerning storage day, leveraging the multiple regression of textural features, with an impressive R-squared value exceeding 0.999.

The study clearly demonstrates that the qualitative characteristics of pastourma begin to exhibit changes between days 8 and 11. Therefore, the study provides strong evidence that the product’s shelf life can be extended by 3 to 5 days beyond the manufacturer’s specifications.

Finally, the overall methodology presented in this work, particularly the non-destructive image analysis technique, has the potential for application in studying other perishable foods. This approach could be used to predict storage durations in terms of quality and safety, providing valuable information for consumers.

## Figures and Tables

**Table 1:** Moisture content (%), hue angle (h), firmness, and TBARS of pastourma samples during storage.

Days of Storage	Day 1	Day 4	Day 8	Day 11	Day 14	Day 16
	**pastourma produced from lamb meat**					
**Moisture content (%)**	45.66 ± 2.86 aA	45.08 ± 3.35 aAΒ	45.54 ± 2.47 aA	37.07 ± 2.58 bA	35.15 ± 3.80 bA	34.93 ± 2.51 bA
**Hue** **angle (h)**	20.45 ± 1.36 abA	20.66 ± 0.65 aA	20.29 ± 1.52 abA	17.33 ± 2.18 bcA	15.84 ± 1.52 cA	14.80 ± 2.02 cA
**Firmness (N)**	5.07 ± 0.62 aA	5.42 ± 0.51 aA	8.56 ± 0.25 bA	9.25 ± 1.21 bcA	11.07 ± 2.35 cA	11.62 ± 1.31 cA
**TBARS (mg malonaldehyde kg^−1^)**	5.10 ± 0.08 aA	5.33 ± 0.22 aA	4.90 ± 0.17 aA	5.31 ± 0.22 aA	5.00 ± 0.15 aA	6.68 ± 0.16 bA
	**pastourma produced from beef meat**					
**Moisture content (%)**	51.62 ± 2.61 aΒ	50.77 ± 3.80 aA	42.18 ± 0.75 bA	42.15 ± 2.80 bB	41.52 ± 2.87 bB	40.37 ± 1.75 bB
**Hue** **angle (h)**	26.42 ± 1.15 abB	27.57 ± 1.56 aB	27.90 ± 1.25 aB	25.83 ± 2.83 abB	25.35 ± 1.79 abB	24.59 ± 1.55 bB
**Firmness (N)**	3.70 ± 0.32 aB	4.12 ± 0.36 abB	5.06 ± 0.61 bB	4.95 ± 0.47 bB	5.19 ± 0.78 bB	5.24 ± 0.59 bB
**TBARS (mg malonaldehyde kg^−1^)**	0.57 ± 0.03 aB	0.66 ± 0.04 bB	0.84 ± 0.04 cB	0.84 ± 0.03 cB	0.86 ± 0.02 cB	0.94 ± 0.03 dB
	**pastourma produced from buffalo meat**					
**Moisture content (%)**	44.23 ± 3.75 aA	39.50 ± 2.53 abB	37.64 ± 2.44 bcB	34.55 ± 2.62 cA	34.18 ± 1.92 cA	35.11 ± 1.93 cA
**Hue** **angle (h)**	23.12 ± 0.99 aC	22.05 ± 2.52 aA	23.99 ± 2.05 aA	22.90 ± 2.14 aB	25.82 ± 3.75 aB	25.16 ± 1.30 aB
**Firmness (N)**	4.48 ± 0.19 aA	5.46 ± 0.24 bA	5.75 ± 0.50 bB	8.87 ± 1.04 cA	9.15 ± 1.80 cA	9.08 ± 1.08 cC
**TBARS (mg malonaldehyde kg^−1^)**	0.97 ± 0.04 aC	1.23 ± 0.05 bC	1.29 ± 0.06 bC	1.47 ± 0.04 cC	1.62 ± 0.05 dD	1.65 ± 0.06 dE

Different low letters in the same row and different capital letters in the same column for the individual parameters indicate statistically different values (*p* < 0.05).

**Table 2:** The spectral absorbance bands (intensities) of pastourma produced from lamb, beef, and buffalo meat during storage.

Days of Storage	Day 1	Day 4	Day 8	Day 11	Day 14	Day 16
**Regions**	**pastourma produced from lamb meat**					
3300 to 3500	0.005 ± 0.001 aA	0.005 ± 0.001 a	0.004 ± 0.002 a	0.005 ± 0.001 a	0.005 ± 0.002 a	0.007 ± 0.001 a
3287–3290	0.218 ± 0.008 aA	0.207 ± 0.007 a	0.199 ± 0.007 a	0.192 ± 0.006 a	0.114 ± 0.006 a	0.088 ± 0.004 b
2950–2960	0.031 ± 0.001 aA	0.027 ± 0.001 a	0.028 ± 0.003 a	0.027 ± 0.002 a	0.028 ± 0.006 a	0.029 ± 0.001 a
2922	0.129 ± 0.008 aA	0.124 ± 0.007 a	0.133 ± 0.011 ab	0.145 ± 0.007 bc	0.159 ± 0.009 c	0.192 ± 0.011 d
2870–2877	0.005 ± 0.001 aA	0.008 ± 0.001 b	0.007 ± 0.001 b	0.008 ± 0.001 b	0.006 ± 0.002 ab	0.008 ± 0.002 b
2854	0.060 ± 0.005 aA	0.036 ± 0.003 b	0.046 ± 0.004 c	0.039 ± 0.002 b	0.073 ± 0.008 d	0.105 ± 0.007 e
1743	0.006 ± 0.001 abA	0.004 ± 0.001 a	0.007 ± 0.001 b	0.012 ± 0.000 c	0.042 ± 0.001 d	0.050 ± 0.001 e
1728	-	-	-	-	0.016 ± 0.005 a	0.031 ± 0.002 b
1627–1630	0.452 ± 0.016 aA	0.442 ± 0.011 a	0.457 ± 0.030 a	0.443 ± 0.013 a	0.432 ± 0.016 a	0.433 ± 0.022 a
1541–1544	0.243 ± 0.014 aA	0.261 ± 0.015 a	0.258 ± 0.013 a	0.257 ± 0.009 a	0.247 ± 0.020 a	0.238 ± 0.021 a
1450–1452	0.039 ± 0.002 abA	0.037 ± 0.001 a	0.045 ± 0.004 b	0.054 ± 0.003 c	0.036 ± 0.002 a	0.032 ± 0.002 d
1392–1400	0.060 ± 0.003 aA	0.059 ± 0.002 a	0.057 ± 0.005 a	0.057 ± 0.002 a	0.037 ± 0.003 b	0.031 ± 0.002 c
1314	0.008 ± 0.001 aA	0.008 ± 0.000 a	0.008 ± 0.001 a	0.009 ± 0.001 a	0.006 ± 0.001 b	0.007 ± 0.001 ab
1238–1242	0.033 ± 0.003 aA	0.030 ± 0.001 a	0.032 ± 0.004 a	0.032 ± 0.001 a	0.034 ± 0.002 a	0.030 ± 0.003 a
1157–1174	0.011 ± 0.002 aA	0.009 ± 0.001 ab	0.008 ± 0.001 b	0.008 ± 0.001 b	0.029 ± 0.002 c	0.025 ± 0.003 c
1078–1083	0.023 ± 0.003 aA	0.024 ± 0.002 a	0.025 ± 0.003 ab	0.029 ± 0.003 bc	0.031 ± 0.002 c	0.037 ± 0.003 d
1060	-	-	0.002 ± 0.000 a	0.010 ± 0.001 b	0.005 ± 0.001 c	0.001 ± 0.000 d
**Regions**	**pastourma produced from beef meat**					
3300 to 3500	0.011 ± 0.001 aB	0.006 ± 0.001 b	0.006 ± 0.001 b	0.004 ± 0.001 b	0.006 ± 0.001 b	0.005 ± 0.001 b
3287–3290	0.170 ± 0.008 aB	0.170 ± 0.007 a	0.191 ± 0.011 b	0.190 ± 0.012 b	0.160 ± 0.006 ab	0.150 ± 0.006 b
2950–2960	0.030 ± 0.003 aA	0.028 ± 0.001 a	0.027 ± 0.001 a	0.027 ± 0.001 a	0.026 ± 0.003 a	0.027 ± 0.001 a
2922	0.053 ± 0.004 aB	0.058 ± 0.002 a	0.066 ± 0.003 b	0.068 ± 0.002 b	0.079 ± 0.004 c	0.086 ± 0.003 d
2870–2877	0.008 ± 0.001 aB	0.008 ± 0.001 a	0.007 ± 0.001 a	0.007 ± 0.001 a	0.007 ± 0.002 a	0.007 ± 0.001 a
2854	0.022 ± 0.003 aB	0.015 ± 0.001 b	0.010 ± 0.002 c	0.031 ± 0.001 d	0.028 ± 0.002 d	0.018 ± 0.001 e
1743	0.024 ± 0.002 aB	0.012 ± 0.001 b	0.006 ± 0.001 c	0.003 ± 0.001 d	0.003 ± 0.001 d	0.004 ± 0.001 d
1728	0.015 ± 0.001 A	-	-	-	-	-
1627–1630	0.487 ± 0.012 aB	0.442 ± 0.011 b	0.444 ± 0.015 b	0.442 ± 0.009 b	0.443 ± 0.017 b	0.440 ± 0.012 b
1541–1544	0.261 ± 0.007 aA	0.256 ± 0.012 ab	0.256 ± 0.009 ab	0.243 ± 0.011 bc	0.241 ± 0.007 c	0.279 ± 0.009 d
1450–1452	0.019 ± 0.002 aB	0.028 ± 0.003 b	0.036 ± 0.002 cd	0.034 ± 0.002 cd	0.032 ± 0.002 c	0.037 ± 0.002 d
1392–1400	0.053 ± 0.004 aA	0.056 ± 0.006 a	0.056 ± 0.002 a	0.055 ± 0.003 a	0.051 ± 0.002 a	0.056 ± 0.003 a
1314	0.008 ± 0.001 aA	0.009 ± 0.001 a	0.009 ± 0.001 a	0.009 ± 0.002 a	0.008 ± 0.001 a	0.009 ± 0.001 a
1238–1242	0.028 ± 0.002 aA	0.033 ± 0.003 b	0.031 ± 0.002 ab	0.030 ± 0.001 ab	0.030 ± 0.003 ab	0.031 ± 0.002 ab
1157–1174	0.009 ± 0.002 abA	0.007 ± 0.001 a	0.009 ± 0.001 ab	0.011 ± 0.002 b	0.009 ± 0.001 ab	0.011 ± 0.001 b
1078–1083	0.027 ± 0.003 aA	0.025 ± 0.002 a	0.018 ± 0.003 b	0.019 ± 0.002 b	0.019 ± 0.003 b	0.012 ± 0.001 c
1060	-	-	-	0.002 ± 0.000 a	0.003 ± 0.000 b	0.008 ± 0.001 c
**Regions**	**pastourma produced from buffalo**					
3300 to 3500	0.004 ± 0.001 aA	0.004 ± 0.001 a	0.005 ± 0.001 a	0.005 ± 0.001 a	0.004 ± 0.001 a	0.004 ± 0.001 a
3287–3290	0.122 ± 0.005 aC	0.115 ± 0.004 a	0.105 ± 0.005 b	0.105 ± 0.004 b	0.104 ± 0.006 b	0.052 ± 0.004 c
2950–2960	0.026 ± 0.002 aB	0.027 ± 0.001 a	0.025 ± 0.003 a	0.027 ± 0.001 a	0.024 ± 0.002 a	0.025 ± 0.002 a
2922	0.052 ± 0.003 aB	0.059 ± 0.002 b	0.070 ± 0.005 c	0.071 ± 0.007 c	0.076 ± 0.003 c	0.051 ± 0.005 a
2870–2877	0.017 ± 0.001 aC	0.017 ± 0.001 a	0.014 ± 0.001 b	0.010 ± 0.001 c	0.007 ± 0.001 d	0.007 ± 0.000 d
2854	0.019 ± 0.001 aB	0.023 ± 0.002 b	0.026 ± 0.002 b	0.031 ± 0.002 c	0.032 ± 0.001 c	0.033 ± 0.001 c
1743	0.020 ± 0.002 aB	0.007 ± 0.001 b	0.006 ± 0.001 b	0.005 ± 0.001 b	0.003 ± 0.001 c	0.002 ± 0.000 c
1728	-	-	-	-	-	-
1627–1630	0.469 ± 0.024 aAB	0.463 ± 0.012 a	0.441 ± 0.014 a	0.449 ± 0.017 a	0.459 ± 0.015 a	0.444 ± 0.018 a
1541–1544	0.257 ± 0.011 aA	0.268 ± 0.011 a	0.257 ± 0.009 a	0.253 ± 0.012 a	0.226 ± 0.009 b	0.232 ± 0.008 b
1450–1452	0.041 ± 0.004 aA	0.034 ± 0.002 b	0.032 ± 0.001 b	0.031 ± 0.002 b	0.033 ± 0.002 b	0.025 ± 0.003 c
1392–1400	0.060 ± 0.004 aA	0.062 ± 0.004 a	0.058 ± 0.002 a	0.060 ± 0.003 a	0.056 ± 0.002 a	0.058 ± 0.003 a
1314	0.008 ± 0.001 aA	0.009 ± 0.001 a	0.008 ± 0.001 a	0.009 ± 0.001 a	0.008 ± 0.002 a	0.009 ± 0.001 a
1238–1242	0.039 ± 0.002 aB	0.031 ± 0.002 b	0.029 ± 0.001 b	0.030 ± 0.002 b	0.028 ± 0.002 b	0.029 ± 0.002 b
1157–1174	0.019 ± 0.001 aB	0.005 ± 0.001 b	0.005 ± 0.001 b	0.007 ± 0.001 b	0.006 ± 0.001 b	0.005 ± 0.001 b
1078–1083	0.026 ± 0.003 aA	0.022 ± 0.003 a	0.014 ± 0.003 b	0.018 ± 0.002 b	0.015 ± 0.002 b	0.026 ± 0.004 a
1060	0.002 ± 0.000 a	0.004 ± 0.001 b	0.007 ± 0.001 c	0.003 ± 0.001 b	0.005 ± 0.001 bc	0.004 ± 0.001 b

Different low letters in the same row and different capital letters in the same column (only day 1) for the individual parameters indicate statistically different values (*p* < 0.05).

**Table 3:** Percentage (%) of protein molecule secondary structure in pastourma samples on day 1 and day 16 of storage.

Protein Molecules Secondary Structure (%)	β-Parallel Sheet (%)	Random Coil (%)	α-Helix (%)	β-Turn (%)	β-Antiparallel Sheet (%)
	**pastourma produced from lamb meat**				
**Day 1**	9.42 ± 0.50 a	18.64 ± 0.35 a	10.16 ± 0.61 a	38.70 ± 1.31 a	23.08 ± 1.08 a
**Day 16**	6.82 ± 0.27 b	13.96 ± 0.34 b	7.05 ± 0.23 b	39.55 ± 1.24 a	32.62 ± 1.39 b
	**pastourma produced from beef meat**				
**Day 1**	7.38 ± 0.82 b	17.68 ± 0.46 c	5.97 ± 0.31 c	31.91 ± 1.12 ce	37.07 ± 1.25 c
**Day 16**	7.03 ± 0.56 b	16.82 ± 0.63 c	8.40 ± 0.56 d	37.89 ± 1.42 a	29.85 ± 1.11 d
	**pastourma produced from buffalo meat**				
**Day 1**	7.06 ± 0.55 b	14.58 ± 0.63 bd	6.57 ± 0.46 cb	29.48 ± 1.09 d	42.30 ± 1.05 e
**Day 16**	6.39 ± 0.69 b	15.59 ± 0.55 d	11.25 ± 0.53 a	33.50 ± 1.49 e	33.26 ± 0.91 b

Different low letters in the same column indicate statistically different values (*p* < 0.05).

## Data Availability

The original contributions presented in the study are included in the article, further inquiries can be directed to the corresponding author.
